# Inflammatory fibroid polyp of the oesophagus

**DOI:** 10.1186/1477-7819-3-30

**Published:** 2005-05-30

**Authors:** Shashi Kanth Godey, Robert T Diggory

**Affiliations:** 1 New Cross Hospital, Wolverhampton, UK; 2 Princess Royal Hospital, Telford, UK

## Abstract

**Background:**

Inflammatory fibroid polyp of the oesophagus is an uncommon lesion and very rarely it grows rapidly.

**Case presentation:**

We present the case of a patient with a rapidly growing inflammatory fibroid polyp (IFP) of the oesophagus, which showed up within five months after a normal endoscopy.

**Conclusion:**

The treatment of IFP is by surgical excision, either open or endoscopic. Laser or other form of ablative treatment like thermo cautery can also be tried.

## Background

Inflammatory fibroid polyp (IFP) is a benign, nonmetastasing tumour of the digestive tract. It has been described to occur commonly in the stomach and intestines. Cases have been reported in rectum [1], duodenum [2] and oesophagus, though rare. It is a slowly growing tumour unlike this case which had rapidly grown in a period of five months. The usual presentation is either with dyspeptic or obstructive symptoms. The treatment is usually confined to local excision of the lesion either endoscopically or by an open procedure. The details of the clinical presentation, histopathological findings and therapeutic choices are discussed.

## Case presentation

A 76-year-old man presented with epigastric pain and dysphagia. Endoscopy revealed grade 1 oesophagitis with a normal stomach and duodenum. Five months later he was readmitted with anorexia, weight loss, dysphagia and anaemia. Endoscopy on this occasion revealed a large pedunculated polypoidal lesion arising from the gastro-oesophageal junction within a hiatus hernia. Pathological examination of biopsies taken from the polyp showed inflammatory changes in squamous mucosa with no evidence of malignancy. As the lesion was pedunculated it was decided to attempt endoscopic snare excision which proved unsuccessful, as the lesion was too large for the snare. At laparotomy a large polypoidal mass was found arising from the distal oesophagus. Through a gastrostomy the lesion was delivered into the abdomen, a linear stapler was applied to the base of the stalk and the lesion was excised. The patient made an uneventful recovery.

**Figure 1:**
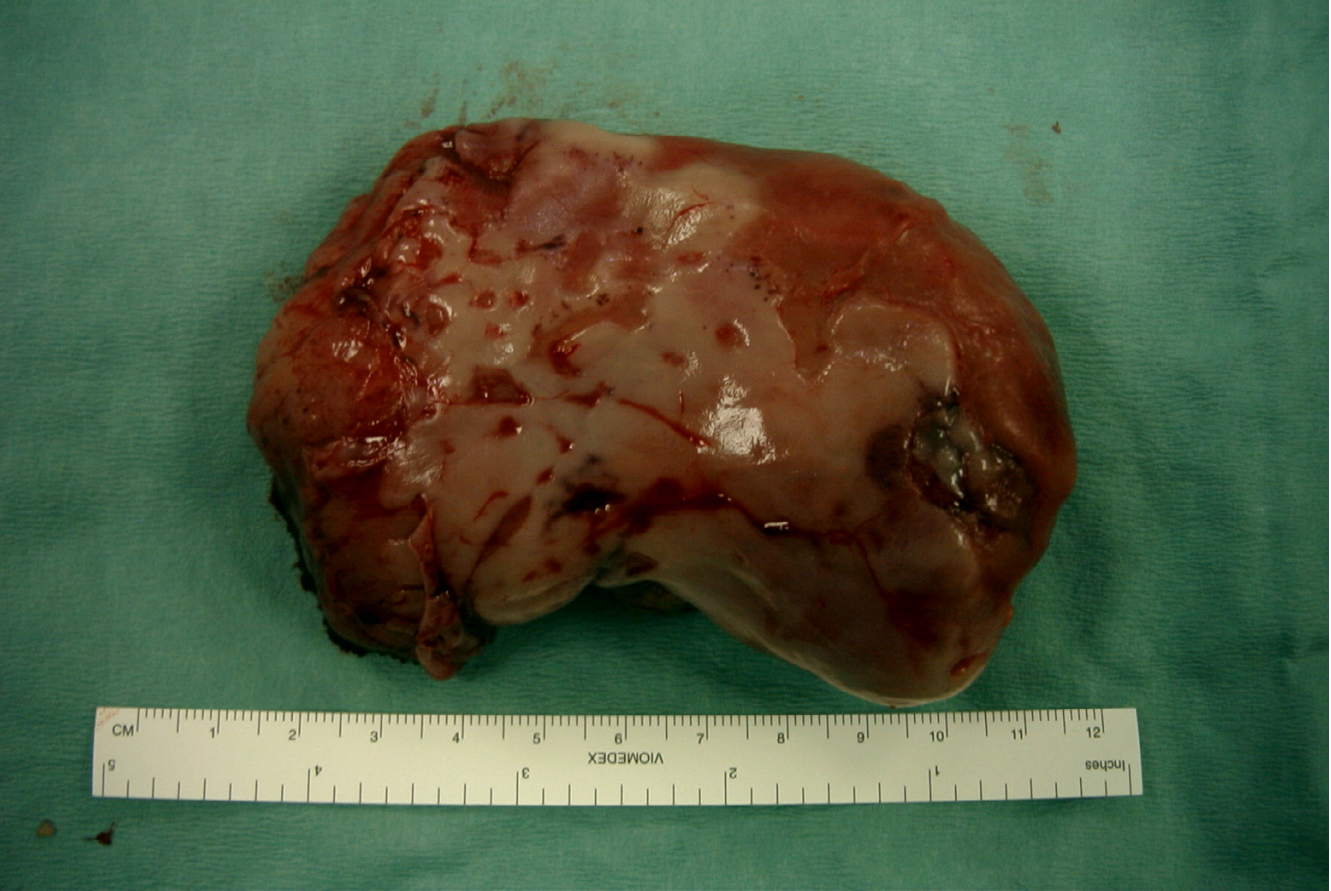
Resected specimen of the polyp measuring 90 × 40 × 40 mm.

**Figure 2:**
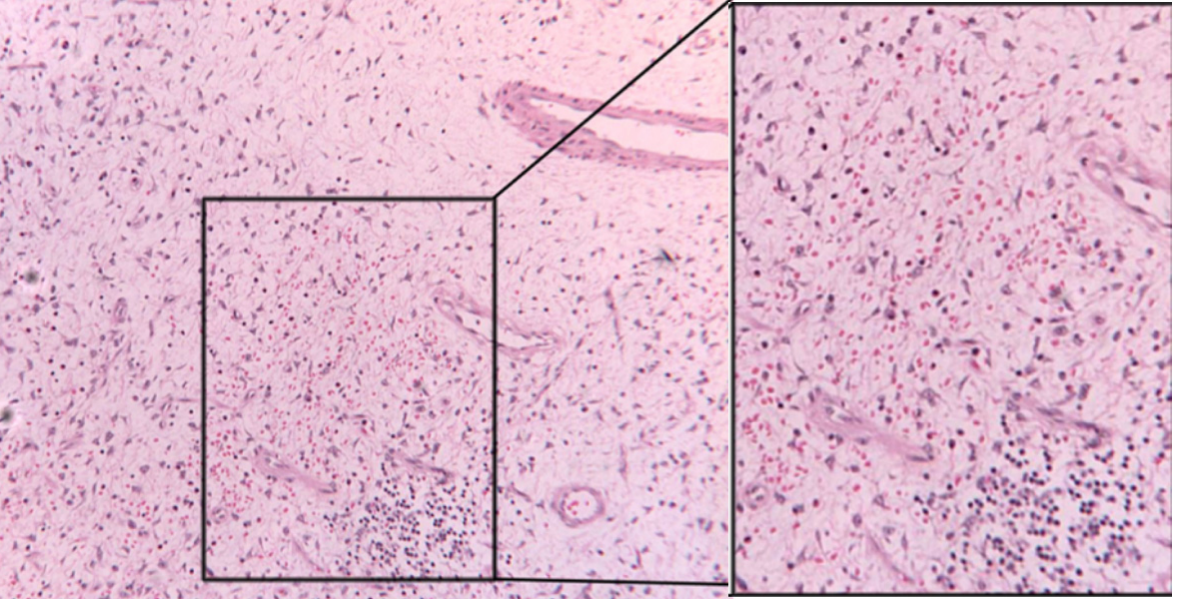
Thick and thin walled blood vessels, stellate fibroblasts, scattered lymphocytes and eosinophils. Shown clearly in the inset view H&E 100.

The resected specimen (figure 1) measured 9 cm × 4 cm × 4 cm and consisted of soft and slimy tissue. Histology showed an intact squamous mucosa and a mass of myxoid fibroblastic tissue with plasma cells and a diffuse eosinophilic infiltrate (figure 2). There was no evidence of malignancy. These appearances were consistent with inflammatory fibroid polyp of the oesophagus. Immunohistochemical studies were negative for S 100 and smooth muscle actin excluding neural or smooth muscle origin.

The patient underwent endoscopy 6 months after resection and there was no evidence of recurrence. He is currently asymptomatic 1 year after resection.

## Discussion

Inflammatory fibroid polyp (IFP) is a rare tumour of the digestive system. It was first described as a distinct pathological entity by J.Vanek in 1949 when he described 6 case reports of gastric submucosal granulomas with eosinophilic infiltration [3]. Since then there have been sporadic case reports but it is difficult to establish the true incidence. The most common site for this lesion in the intestinal tract is the stomach where an incidence of 4.5% of all gastric polyps has been reported [4,5]. Our patient had an oesophageal lesion which is an unusual location although there have been 9 reported cases studies in the literature [6-12]. It usually occurs as a solitary lesion, the distal 1/3^rd ^of the oesophagus being the commonest site for this tumour. Inflammatory fibroid polyp is a benign lesion of uncertain origin [7] some reports claiming it to be myofibroblastic [13] in origin and others suggest it arises from vascular or perivascular tissue [14]. The main characteristics of this lesion are eosinophilic infiltration and presence of characteristic connective tissue stroma. It is generally accepted that this is not a neoplasm, but is reactive process [8] to physical, chemical or microbiological stimuli.

The presentation is dependant on the size, location or complications of the tumour. In those of the stomach, epigastric pain and bleeding are the common symptoms and those of the intestine [15] colicky abdominal pain is the most common symptom. In the oesophagus it can present with dysphagia, bleeding or gastro-oesophageal reflux symptoms [6,7,11,16,17]. The clinical presentation in our case was with progressive dysphagia and melaena. IFP has a submucous location and grows intraluminally. There is no reported evidence of metastatic spread in literature. It is difficult to differentiate IFP from the other lesions that occur in the oesophagus like leiomyoma, lipoma, pedunculated papilloma, fibrovascular polyp. The definitive diagnosis is histological. Histological differentiation is from fibrovascular polyp which shows mature fibrous tissue with scattered thin-walled blood vessels lined by flat non reactive endothelial cells with most of them containing fat. Differentiation from leiomyoma is based on the finding that bundles of nonneoplastic smooth muscle can be seen at the level of pre-existing muscularis propria in case of an IFP.

The patient was initially evaluated with an endoscopy, which showed grade1 oesophagitis. Subsequent endoscopy localised the lesion as arising from the distal oesophagus near to the gastro-oesophageal junction, and had a benign appearance. The discovery of the lesion on endoscopy within five months of a previous normal endoscopy does give an indication that it may be rapidly growing [18]

The previously reported studies showed that the usual presentation of IFPs varied from months to years, but we could not attribute any cause for the rapidity of growth of this lesion in our patient. Investigation modalities that are used for these lesions include radiographs, endoscopic ultrasound, CT scan and MRI. In our case in view of the acute presentation these were deferred.

The treatment is confined to surgical excision of the lesion by endoscopic or open method, the decision based on the site and size of the tumour. There are cases which had to be resected by a lateral oesophagotomy or formal oesophagectomy [12]. There has also been reported use of Thermocautery and Nd YAG Laser in the treatment of small polyps. Our patient had an initial attempt of resection of the tumour endoscopically but in view of its size it had to be abandoned and subsequently resected after gastrostomy.

## Conclusion

The commonest site of IFP in the GI tract is stomach; IFP of the oesophagus is rare. Treatment is by excision that can be carried out endoscopically. LASER ablation and coagulation has been tried.

## Competing interests

The author(s) declare that they have no competing interests.

## Authors' contributions

**SKG**- wrote the original manuscript, review of literature, prepared requested revisions

**RTD**- performed surgical resection, helped to draft the manuscript.

All authors read and approved the final manuscript.
